# Acceptability of IV iron treatment for iron deficiency anaemia in pregnancy in Nigeria: a qualitative study with pregnant women, domestic decision-makers, and health care providers

**DOI:** 10.1186/s12978-024-01743-y

**Published:** 2024-02-13

**Authors:** Opeyemi R. Akinajo, Ochuwa A. Babah, Aduragbemi Banke-Thomas, Lenka Beňová, Nadia A. Sam-Agudu, Mobolanle R. Balogun, Victoria O. Adaramoye, Hadiza S. Galadanci, Rachel A. Quao, Bosede Bukola Afolabi, Kristi Sidney Annerstedt

**Affiliations:** 1 Department of Obstetrics and Gynaecology, Lagos University Teaching Hospital, Idi-Araba, Lagos, Nigeria; 2 Department of Global Public Health, Karolinska Institutet, Stockholm, Sweden; 3 Department of Public Health, Institute of Tropical Medicine, Antwerp, Belgium; 4 Department of Obstetrics and Gynaecology, Faculty of Clinical Sciences, College of Medicine, University of Lagos, Idi-Araba, Lagos, Nigeria; 5 Maternal, Adolescent, Reproductive and Child Health (MARCH), Centre, London School of Hygiene and Tropical Medicine, London, UK; 6 International Research Center of Excellence, Institute of Human Virology Nigeria, Abuja, Nigeria; 7 Institute of Human Virology, University of Maryland School of Medicine, Baltimore, USA; 8 Department of Community Health and Primary Care, College of Medicine, University of Lagos, Lagos, Nigeria; 9 African Center of Excellence for Population Health and Policy, Bayero University, Kano, Nigeria; 10 Department of Obstetrics and Gynaecology, College of Health Sciences Bayero University Kano/ Aminu Kano Teaching Hospital, Kano, Nigeria; 11 The Centre for Clinical Trials, Research, and Implementation Science (CCTRIS), University of Lagos, Idi-Araba, Lagos, Nigeria

**Keywords:** Anaemia, Iron deficiency anaemia, Pregnancy, Acceptability, Intravenous iron, Oral iron, Implementation science, Maternal health, Perinatal health, Maternal mortality, Maternal morbidity

## Abstract

**Background:**

Anaemia in pregnancy causes a significant burden of maternal morbidity and mortality in sub-Saharan Africa, with prevalence ranging from 25 to 45% in Nigeria. The main treatment, daily oral iron, is associated with suboptimal adherence and effectiveness. Among pregnant women with iron deficiency, which is a leading cause of anaemia (IDA), intravenous (IV) iron is an alternative treatment in moderate or severe cases. This qualitative study explored the acceptability of IV iron in the states of Kano and Lagos in Nigeria.

**Methods:**

We purposively sampled various stakeholders, including pregnant women, domestic decision-makers, and healthcare providers (HCPs) during the pre-intervention phase of a hybrid clinical trial (IVON trial) in 10 healthcare facilities across three levels of the health system. Semi-structured topic guides guided 12 focus group discussions (140 participants) and 29 key informant interviews. We used the theoretical framework of acceptability to conduct qualitative content analysis.

**Results:**

We identified three main themes and eight sub-themes that reflected the prospective acceptability of IV iron therapy. Generally, all stakeholders had a positive affective attitude towards IV iron based on its comparative advantages to oral iron. The HCPs noted the effectiveness of IV iron in its ability to evoke an immediate response and capacity to reduce anaemia-related complications. It was perceived as a suitable alternative to blood transfusion for specific individuals based on ethicality. However, to pregnant women and the HCPs, IV iron could present a higher opportunity cost than oral iron for the users and providers as it necessitates additional time to receive and administer it. To all stakeholder groups, leveraging the existing infrastructure to facilitate IV iron treatment will stimulate coherence and self-efficacy while strengthening the existing trust between pregnant women and HCPs can avert misconceptions. Finally, even though high out-of-pocket costs might make IV iron out of reach for poor women, the HCPs felt it can potentially prevent higher treatment fees from complications of IDA.

**Conclusions:**

IV iron has a potential to become the preferred treatment for iron-deficiency anaemia in pregnancy in Nigeria if proven effective. HCP training, optimisation of information and clinical care delivery during antenatal visits, uninterrupted supply of IV iron, and subsidies to offset higher costs need to be considered to improve its acceptability.

*Trial registration* ISRCTN registry ISRCT N6348 4804. Registered on 10 December 2020 Clinicaltrials.gov NCT04976179. Registered on 26 July 2021

## Plain language summary

Low blood level in pregnancy is of public health importance and with common occurrence worldwide, but with a higher rate in low resource settings where its burden greatly affects both the mother and her baby. This low blood level is usually caused by poor intake of an iron-rich diet. It could lead to fatigue, decreased work capacity, and dizziness if not detected. Without treatment, this condition could affect the baby, possibly leading to its sudden demise in the womb, immediately after birth, or even the woman’s death.

The use of oral iron has been the primary treatment; however, it is associated with significant side effects, which have led to poor compliance. Fortunately, an alternative therapy in the form of a drip has been shown to overcome these challenges. However, it is not routinely used in countries like Nigeria. Moreover, being effective is different from being utilised. Therefore, this study was conducted to understand the factors that will make this treatment widely accepted.

We interviewed pregnant women, family support and health care providers in 10 health facilities in Lagos and Kano States, Nigeria. Our findings revealed good attitudes to iron drip. However, its inclusion into routine antenatal health talk, training of health care providers, availability of space, drugs and health workers who will provide this care, and ensuring this drug is of low cost are some of the efforts needed for this treatment to be accepted.

## Background

Anaemia in pregnancy (AIP) is a condition of public health significance with a prevalence of 36% globally, 41% in Africa, [1] and 25–45% in Nigeria [2]. Iron deficiency anaemia (IDA) accounts for 50–75% of AIP and is caused by insufficient iron intake and/or absorption and demand from the growing foetus [2, 3]. Undiagnosed and untreated IDA can result in extreme fatigue, reduced physical and mental function [4, 5], and depression [6]. It also increases the risk of postpartum haemorrhage (PPH) [7], which, together with anaemia, contributes to 40–43% of maternal mortality in Africa and Asia [8]. Furthermore, it is associated with twice as high maternal mortality in women with severe anaemia than those without [9]. Additionally, IDA has been linked to poor pregnancy outcomes such as intrauterine growth retardation, premature birth, low infant birth weight, and stillbirths in severe cases [4, 5].

The mainstay treatment modality that the World Health Organization (WHO) recommends for managing IDA in pregnancy is daily oral iron therapy, a low-cost treatment [10]. However, over 70% of these pregnant women are unable to fully benefit from this intervention due to significant gastrointestinal adverse effects leading to poor tolerance and suboptimal adherence [11–13]. Intravenous (IV) iron is an alternative treatment indicated for pregnant women with moderate to severe IDA in several high-income countries (HICs) [14, 15]. In these HICs and increasingly in low- and middle-income countries (LMICs), IV iron administered as a single, rapid infusion is preferable to the shortcomings of daily oral iron therapy based on its safety profile and effectiveness [16, 17]. However, establishing the effectiveness of this treatment alone does not guarantee its implementation or uptake into routine clinical use in LMICs. Successful implementation of such intervention also depends on its acceptability to both those providing and receiving the intervention [18].

To support any future practice or policy change toward the routine provision of IV iron for pregnant women with IDA in LMICs, it is necessary to understand its acceptability. Such understanding could pave the way for its rapid uptake and aid in developing standard protocols for care and full integration into routine practice. However, there is limited evidence on the acceptability of IV iron therapy in LMICs, especially in sub-Saharan Africa [19]. To address this evidence gap, the objective of this study was to prospectively understand the acceptability of IV iron therapy among a range of stakeholders in Nigeria.

## Methods

### Study design

This is an exploratory qualitative phenomenological study [20, 21] nested within the context of an open-label hybrid type 1 effectiveness-implementation trial of IV versus oral iron for Iron Deficiency Anaemia in Pregnant Nigerian Women (IVON) [22]. The hybrid trial design allowed for the exploration of the acceptability of IV iron as part of formative research among prospective users and domestic decision-makers as family support and healthcare providers (HCPs). We report the study following the consolidated criteria for reporting qualitative research (COREQ) [23].

### Study setting

Nigeria has > 200 million inhabitants and an estimated seven million births yearly [24]. Less than two-thirds of pregnant women in Nigeria complete four or more antenatal care (ANC) visits, and 60% give birth outside of health facilities [24]. Nigeria’s maternal mortality ratio is 1047 per 100,000 live births, accounting for more than a quarter (28%) of all maternal deaths globally [25]. This study was conducted in IVON trial sites in Nigeria’s two most populous states: Kano, in the North-Western part of the country, and Lagos, in the South-West [26]. These states were selected for the IVON trial based on the differences in uptake, coverage, and use of maternal health services. For example, ANC utilisation rates were 63% in urban Kano, 51% in rural Kano and 81% in Lagos [27, 28] The percentage of births assisted by skilled health personnel (18% in the North-West and 85% in the South-West) [24] and the prevalence of AIP as seen in different studies (from 7 to 75% in Kano and from 35 to 87% in Lagos) [29–32]. Our study was conducted in ten health facilities across the three levels of the Nigerian health system (two primary health centres [PHCs], two secondary hospitals, and one tertiary hospital per state) [33, 34]. During data collection for this study, the facilities were in the process of being onboarded as IVON trial sites. As such, all had ANC clinics serving at least 60 pregnant women per month, onsite testing for haemoglobin, human immunodeficiency virus (HIV) and malaria, and a labour ward which operates 24 h/day and conducts at least 20 deliveries per month.

### Recruitment procedure

We used purposive and snowball sampling techniques [35], which enabled the identification of key stakeholders: pregnant women, domestic decision-makers (male partners and matriarchs such as mothers and mothers-in-law of pregnant women), various cadres of health care providers (HCPs) who would administer IV iron (nursing officers, nurse-midwives, medical officers, including consultants and resident doctors specialising in obstetrics) and health system decision-makers (health facility managers such as medical directors and apex HCPs, e.g., heads of departments, principal medical officers, chief nursing officers, chief pharmacists, and laboratory scientists).

We recruited pregnant women attending ANC clinics in the ten study facilities. The following women’s characteristics were used to purposively sample: age, parity, gestational age, education, and previous/current diagnosis of AIP based on the assumption that these factors could influence the acceptability of IV iron. HCPs who provided antenatal care services, apex HCPs and health facility managers were recruited from the ten facilities. The HCPs included males and females, various cadres, and various years in service. Male partners and matriarchs were recruited from the communities close to the selected healthcare facilities through referrals from the ward health committee leaders, who oversee the planning, organisation and mobilisation of resources and people for health programs in the community. We aimed for a sample size with considerations of high information power [36] based on the study aim, sample specificity involving different stakeholders in multiple centres in two states and the strong quality of dialogue from various points of view.

### Data collection

Eight interviewers trained in qualitative data collection methods facilitated focus group discussions (FGDs) and key informant interviews (KIIs) among selected study participants. We chose FGDs for pregnant women in general (with or without anaemia), HCPs, male partners, and matriarchs because the interaction of participants within FGDs provided the most robust description of their perception of acceptability [37, 38]. KIIs for pregnant women with anaemia, apex HCPs and facility managers were selected as they provided an opportunity for one-on-one in-depth discussions and were more practical for many of these study participants [37, 38]. Each FGD had three research team members present; one to facilitate the FGD, one to monitor the process and record the interview, and a third who took notes on the discussion generally, including non-verbal signs and emotions, such as facial expressions. Each KII had two team members, one to conduct the interview and the other to monitor and record. Interviews with HCPs, apex HCPs, health facility managers, and literate pregnant women were conducted in English. In addition, interviews in Yoruba and Hausa languages were conducted based on the preferred language as decided by the other study participants (male partners, matriarchs and some pregnant women who were not educated). All interviews (FGDs and KIIs) were conducted in the ten health facilities selected for the study. We ensured that participants felt comfortable with their environment to foster a calm and positive atmosphere and build a rapport between the research team and the participants [38].

All FGDs and KIIs were conducted with semi-structured topic guides. These guides were designed using the Consolidated Framework for Implementation Research (CFIR) to elicit an understanding of the experience of AIP, the perception of existing oral therapy and the acceptability of IV iron therapy. All topic guides were written in English and later translated into Hausa and Yoruba languages (forward translation) and back-translated into English language. Each FGD lasted between 60 and 90 min, and KII between 30 and 45 min. We invited 176 participants to the FGDs, of which 36 declined. For the KIIs, out of the 39 participants invited, ten declined. In total, we conducted 12 FGDs among 140 participants and KIIs among 29 participants who voluntarily consented to be interviewed between April 6 and May 7, 2021 (Table 1). The tools were piloted ahead of data collection and adjusted where appropriate for clarity. All the audio-recorded interviews were transcribed verbatim and, where necessary, translated and transcribed into English (from Yoruba or Hausa).

**Table 1:** Summary profiles of participants interviewed in this study by method

Participant group		No. FGDs/KIIs			No. participants		
		Lagos	Kano	Total	Lagos	Kano	Total
Focus Group Discussions (FGDs)							
1	Pregnant women with or without anaemia	2	2	4	27	20	47
2	Matriarchs	1	1	2	12	12	24
3	Male partners	1	1	2	11	12	23
4	HCPs	2	2	4	24	22	46
Total number of FGDs		6	6	12	74	66	140
Key Informant Interviews (KIIs)							
1	Pregnant women with anaemia	5	5	10	5	5	10
2	Apex HCPs	8	7	15	8	7	15
3	Health Facility Managers	2	2	4	2	2	4
Total number of KIIs		15	14	29	15	14	29
Total		21	20	41	89	80	169

Heads of departments, principal medical officers, chief nursing officers, chief pharmacists, and laboratory scientists are stakeholders referred to as Apex HCPs. Medical directors are facility managers

### Data analysis

The theoretical framework of acceptability (TFA) by Sekhon et al. was used to inform and guide the analysis [18]. This multi-faceted framework has seven constructs: affective attitude, burden, ethicality, intervention coherence, opportunity costs, perceived effectiveness, and self-efficacy (Table 2). TFA allowed us to capture the important dimensions of the prospective acceptability of IV iron therapy and a joint analysis of the varied stakeholders as the receiver, support, or provider of this intervention. We analysed our dataset using content analysis as it allowed a flexible approach to identify, analyse, and systematically organise the data into a structured format [39]. Familiarisation with the dataset was done, meaning units were identified, condensed, and coded, and an initial coding framework was developed deductively using the predetermined constructs of the TFA framework. Next, two qualitatively trained researchers (ORA & AB-T) randomly selected three transcripts and coded them independently. The codes were discussed, and a final coding framework developed. ORA then coded the remainder of the data. All codes were subsequently arranged into categories, after which key themes and sub-themes emerging from the data were generated [40, 41]. The emerging themes and sub-themes were subsequently discussed with other research team members (ORA, AB-T, KSA, LB, BBA) and their descriptions refined as deemed necessary. NVivo 12 Plus (QSR International, Memphis, USA) aided analysis.

**Table 2:** Theoretical framework of acceptability of IV iron adapted from Sekhon et al.’s seven constructs

Constructs	Definition
Affective attitude	How an individual (participant) feels about IV iron
Burden	The perceived amount of effort that is required to receive or administer IV iron
Ethicality	The extent to which IV iron has a good fit with an individual’s (participant’s) value system
Intervention coherence	The extent to which participants understand IV iron and how it works
Opportunity cost	The extent to which benefits profits, or values must be given up to receive or administer IV iron
Perceived effectiveness	The extent to which IV iron is perceived to be likely to achieve its purpose
Self- efficacy	The participants’ confidence that they can perform the behaviour(s) required to receive or administer IV iron

### Ethical considerations

Ethical approval was obtained from the National Health Research Ethics Committee of Nigeria (NHREC/01/01/2007- 17/01/2021), Health Research and Ethics Committees of the Lagos University Teaching Hospital (ADM/DCST/HREC/APP/3971), Lagos State Health Service Commissions (LSHSC/2222/VOLIII), Lagos State Primary Health Care Board (LS/PHCB/MS/1128/VOL.VII/100), Aminu Kano Teaching Hospital, Kano State (NHREC/28/01/2020/AKTH/EC/2955) and Ministry of Health, Kano State (MOH/Off/797/T.1/2102). In addition, information on voluntary participation, privacy, confidentiality, risks, and benefit were provided to all participants and obtained consent to participate in the study. Audio recording commenced only after obtaining permission verbally from the participants.

## Results

We identified three main themes around the acceptability of IV iron: perceived comparative advantages of IV iron over oral therapy; existing infrastructure in the health facility which could be leveraged and strengthened to sustainably provide IV iron; and existing high-level of trust between pregnant women and HCPs which can avert potential misconceptions about IV iron therapy. Eight sub-themes described factors associated with the acceptability of IV iron therapy (Table 3). These themes and sub-themes were mapped to all domains of TFA.

**Table 3:** Illustrating identified themes and sub-themes mapped into seven constructs of the TFA

Theme	Sub-theme	TFA construct	Stakeholders to whom this theme was relevant
1. Perceived comparative advantages of IV iron are critical for acceptability	1.1. For iron supplementation, anything is better than taking pills	Affective attitude Perceived effectiveness Opportunity cost	Pregnant women, domestic decision-makers, and HCPs
	1.2. Reduction of anaemia-related complications could ease HCP workload	Opportunity cost Perceived effectiveness	HCPs
	1.3. Preferred alternative to blood transfusion	Affective attitude Ethicality	HCPs and apex HCPs
2. Existing infrastructure in the health facility could be leveraged and strengthened to sustainably provide IV iron	2.1. Existing processes of care provision to integrate information on IV iron therapy into antenatal health talk sessions	Burden Intervention coherence Self-efficacy	Pregnant women, domestic decision-makers, and HCPs
	2.2. HCPs lack confidence but are optimistic to safely administer IV iron with further training	Burden Self-efficacy	Pregnant women, domestic decision-makers, HCPs and apex HCPs
	2.3. Local health system infrastructure, resources and supplies are insufficient	Burden	HCPs and facility managers
	2.4. High out-of-pocket costs might make IV iron out of reach for the most vulnerable and socio-economically disadvantaged women	Burden Perceived effectiveness	Pregnant women, domestic decision-makers, and HCPs
3. Existing trust between pregnant women and HCPs can avert misconceptions of IV iron therapy	3.1. Pregnant women trust HCPs, but vulnerable to misconceptions	Burden	Pregnant women, domestic decision-makers, and HCPs

*TFA*, theoretical framework of acceptability, *HCP* health care provider, *IV* intravenous

### Perceived comparative advantages of IV iron are critical for acceptability

All stakeholder groups identified some advantages of IV iron therapy over oral treatment, which are critical for its acceptance by both users and the HCPs providing IV iron. These are the advantages IV iron has over oral iron and blood transfusion, including its capacity to reduce workload among HCPs.

#### For iron supplements, anything is better than taking pills

Considering the challenges with oral iron therapy, such as intolerance, the burden of daily use throughout pregnancy until the postpartum period and gastrointestinal side effects, pregnant women, HCPs, and domestic decision-makers believed that any other iron formulation was better than ‘pills’ to treat IDA. When discussing alternatives to oral iron therapy, they perceived IV iron as a ‘*perfect*’ treatment. They described several advantages, including prompt response to treatment, being a good fit for patients with oral iron intolerance and those irritated by the taste and or smell of oral iron, and overall, improving treatment adherence. A pregnant woman who had experience with taking oral in previous pregnancy for IDA and hardly tolerated food from excessive vomiting stated her preference for IV iron therapy.

*“If IV iron is available, I will receive it because I used to have complications like excessive vomiting. I also do not tolerate food during pregnancy and usually have low blood levels. So, anything that will help me to get better, I will look for it, honestly.” 32-year-old, Multigravida, Kano (KII-04)*

The opinion above was acknowledged by HCPs, who explained that excessive vomiting in pregnancy, including intolerance to oral iron—whether tablet or suspension—was not only of concern to pregnant women but also to the HCPs who provide care to them and feel IV iron was a suitable alternative. To further emphasise this point, one of the HCPs, a specialist trained in obstetrics and gynaecology who had previously administered IV iron to a severely ill patient described the “*immediate improvement in health*.” He also felt that “*not repeatedly coming to the facility for more doses was a big advantage compared to oral iron*.”

Furthermore, pregnant women, HCPs and domestic decision-makers highlighted that they believed IV iron could solve the issues of adherence to oral iron. “*Lack of will, forgetfulness and the burden of daily use of oral pills throughout pregnancy and after delivery*” were highlighted by pregnant women as the cause of poor adherence. Therefore, these pregnant women will “*choose IV iron therapy over the oral formulation because it will improve adherence to the treatment of IDA, more importantly, because of its one-time use throughout pregnancy*.” This opinion was also reflected in the response of a male partner from Kano who stated that *“if I do not get involved with the routine use of oral iron for my wife, she will never take it,”* hence his preference for IV iron. For the HCPs, being an HCP-dependent intervention makes IV iron a superior choice which comes with the assurance of its adherence, unlike the oral route. However, from the perspective of a pregnant woman who had received IV iron previously, to receive this therapy meant coming to the facility, which is an additional effort compared to the oral route.

*“Anyway, the thing with IV iron is that it works, but l have to come in, and this is different from the pills that can be taken at home. l have to be monitored... and it could take an hour or two for the whole process”. 35-year-old, Multigravida with anaemia, Lagos (KII- 02)*

#### Reduction of anaemia -related complications will ease HCP workload

According to the HCPs, even though administering IV iron means increased workload as time spent caring for patients increases, it is an excellent option to yield immediate response to treatment, treat IDA, and reduce the risk of anaemia-related complications. Some HCPs also believed that IV iron would reduce the rate of feto-maternal complications. According to some other HCPs, it will ultimately reduce the overflow of the workload associated with the high prevalence of IDA.

*“So, this intravenous iron will reduce the number of patients with anaemia and all its complications, so it is very effective or useful for healthcare workers.” Apex HCP, Male, Kano (FGD P6)*

Furthermore, according to the HCPs, IV iron could generate a positive outcome for both the mother and the baby and achieve the desired outcome of all HCPs, which is to reduce the rate of maternal and perinatal morbidity and mortality associated with IDA.

#### Preferred alternative to blood transfusion

The HCPs felt that IV iron could help avert blood transfusions needed to treat complicated IDA during and after birth, which had many significant benefits. According to them, IV iron administered on an outpatient basis in 15 to 20 min could promptly treat IDA and reduce the need for a blood transfusion that can take several hours or days. In addition, they believed that IV iron was more cost-effective compared to blood transfusion. For some HCPs, *“IV iron can avert the cost of screening for blood-borne transmitted infections, consumables, and admission for blood”*. According to some other HCPs, it can also avert problems associated with blood transfusion such as challenges of getting donors to donate blood, and the lack of compatible blood in hospitals. Most HCPs also felt that IV iron is a suitable alternative to blood transfusions before its need arises for specific individuals based on their beliefs and values. They gave examples of some pregnant women who delay or refuse to receive blood transfusions based on their personal or religious beliefs, thereby increasing their risk of morbidity or mortality.

### Existing infrastructure in the health facility could be leveraged and strengthened to sustainably provide IV iron

Participants in each stakeholder group agreed that the maternal healthcare system has an existing infrastructure that could be leveraged to guarantee the acceptability of IV iron and strengthened for sustenance. While it is not yet designed to effectively deliver IV iron therapy, these participants stated that the existing processes of care provision could integrate information on IV iron into ANC health talks, train HCPs and ensure constant resources and supplies.

#### Existing processes of care provision to integrate information on IV iron therapy into ANC health talk sessions

As reported by all stakeholder groups, there are existing well-functioning processes in place to provide care, which include information sharing and education for pregnant women in the form of group health talks during all ANC visits. According to them, information on IV iron can be integrated into the existing ANC services and modified to promote it as an option for managing IDA. For example, one of the participants who had received IV iron outside of Nigeria during her previous pregnancy attributed adequate counselling to her positive perception and, ultimately, acceptance of the therapy. Furthermore, she believed pregnant women would accept IV iron if HCPs could build on the existing ANC health talks to include discussions on the importance of adequate treatment of IDA in pregnancy with IV iron.

*“Pregnant women will accept it if it is explained to them; you know if the difference is explained to them. They will ask you why you are bringing an IV [iron] since they already have the oral, so if the health care workers explain that this IV [iron] increases, makes it easy and even improves the anaemia drastically, it will make a difference. l feel anything that will protect the health of women and prevent death, they will accept it. You know we already have our health talk, which has helped educate our women, so we should use this to educate them on the importance of IV iron when talking to them in the antenatal clinic.” 35-year-old, Multigravida with anaemia, Lagos (KII- 02)*

Another pregnant woman whose baby died during birth from complications of IDA stated that the HCPs should include IV iron as a treatment option in their health talks to warn pregnant women about the dangers of untreated IDA. The importance of this was further elucidated by the HCPs, who stated that the demand for treating IDA with IV iron would increase if the focus were on how IV iron works, the process of administration, and its health benefit to the individual and the baby. Furthermore, according to them, it could facilitate a deeper understanding and knowledge of pregnant women and enhance decision-making.

#### HCPs lack confidence but are optimistic to safely administer IV iron with further training

The HCPs stated that IV iron administration needs to be monitored closely, given the risk of adverse effects, thus necessitating the need for training before it is provided routinely. In addition, some HCPs lacked the confidence to respond promptly and accurately in the event of severe reactions. This concern was expressed by some HCPs from primary healthcare facilities where only basic healthcare services are typically performed. To them, IV iron is indicated in severe anaemic cases and should be referred for administration in a secondary health facility with the capacity for rapid response and immediate treatment. Generally, there was consensus among the HCPs with eagerness and readiness to administer IV iron therapy, however, they highlighted the need for specialised training and a protocol to facilitate safe administration and identification of reaction symptoms.

*“Training is crucial…. For IV iron, we need to be able to give it with confidence. Because with training on how to give safely, we become more confident and more efficient. So, health workers’ training is important.” Apex HCP, Male, Lagos (KII-08)*

To pregnant women and domestic decision-makers, it would be reassuring if IV iron were administered by personnel and teams trained to give it.

#### Local health system infrastructure, resources and supplies are insufficient.

Challenges such as inadequate physical space, limited human resources, and poor supply chain were reported by HCPs and facility managers as having significant potential to affect the acceptability of IV iron. According to these respondents, inadequate availability of space, especially in facilities with many patients and in primary health care facilities, is of concern. In addition, as a new treatment requiring close patient observation, they believed that dedicated spaces should be allocated explicitly for IV iron to facilitate a smooth administration process for the HCPs and pregnant women. For some HCPs, adequate space to administer IV iron would be the only foreseen challenge they identified in their facility.

In addition, HCPs stated that the lack of human resources is a challenge affecting the existing maternal care in most facilities in the country, considering the high volume of patients and workload in general. For example, one of the nurses explained that IV iron administration would be an additional burden to HCPs if there were no provision for adequate human resources to assist with this intervention. In addition, many questioned the feasibility of administering IV iron as they expressed their fears based on the volume of work and patients they see daily. According to a HCPs in a KII, “*IV iron is a personnel-dependent form of care compared to the oral iron formulation, which entails close monitoring to prevent, identify and promptly respond to any allergic reactions”*. And hence, it *“makes it more challenging for IV iron to be readily acceptable among HCPs”*.

Given their experience with frequent essential medicine and supply stockouts in the public sector, HCPs were concerned that they could not consistently offer this service if such issues also affected IV iron. In an FGD, one of the HCPs stated:

*“The challenge we could have as health workers is the availability of medications because sometimes when we introduce a medication, we will get the supply for some time but later go out of stock. Then we are not able to get it anymore. So, the biggest challenge we will have is the irregular supply of this IV drug.” HCP, Female, Lagos (FGD P12)*

Additionally, the availability of essential equipment and commodities, as stated by some of the HCPs, is necessary to provide quality maternal care, including when receiving IV iron. Therefore, the HCPs suggested creating a package where all the required commodities are in one place to facilitate easy access.

*“We need to create a pack for it just like a pack for TIVA (Total intravenous anaesthesia), which contains everything, including the cost. So, the process will be to create the pack for this intervention and take it to where it is needed… We must remember that it is a result-oriented medical therapy compared to some of the other options.” HCP, Male, Lagos (FGD P1)*

#### High out-of-pocket costs might make IV iron out of reach for the most vulnerable and socio-economically disadvantaged women

Due to the high prevalence of poverty in the country, most participants felt the cost of IV iron therapy could significantly impact the perception and use from the women’s and providers’ perspectives. This became more evident when participants compared IV to oral iron. They stated that while oral iron is potentially less effective, it is typically dispensed free of charge or given at a subsided cost. According to pregnant women, if IV iron is not available at a comparably low-cost, oral iron will be preferred. For most male partners, even though they will support and encourage their partners to take IV iron if it is available, they “*hope God will provide*” for them to afford it.

The HCPs voiced their concern regarding the type of pregnant women more likely to have anaemia, which are more likely to be women from low socioeconomic classes who reside mostly in rural areas. According to them, these women cannot even afford to register for routine ANC services, buy essential drugs or pay for routine tests. If IV iron is not heavily subsidised or free, poor women who are most likely affected by IDA will be unable to use it. The cost of IV iron will be the predominant acceptability factor for women and their households.

*“One thing you did not tell us that may serve as a hindrance is whether it will be given free or they will pay for it. The price will determine its acceptance because, as you know, we are in an economic crisis…Let us be realistic, its acceptance will depend on the price. Because the husbands are usually poor sometimes, they cannot even afford to buy common drugs. Some of them find it hard to even pay for the initial investigations… Not every man can afford that.” HCP, Female, Kano (FGD P1)*

According to the HCPs, beyond the cost of the IV iron itself are the additional costs of administering it (consumables, intravenous fluids etc.) which need to be considered. Therefore, in their opinion, most pregnant women affected by IDA will likely be financially constrained to utilise this effective treatment.

*“l think having a subsidy on this is not out of place because if you look at the health insurance scheme at the moment is not yet robust. We are talking about anaemia…it is one of the contributors to maternal mortality. So now, if you want to wait until the national health insurance scheme is very effective to take care of it without subsidy, what happens to that woman in [urban slum] selling fish or the woman in [slum settlement in Lagos] selling water? Then there will be a challenge. These are the people that come down with anaemia during pregnancy.” HCP, Male, Lagos (FGD P5)*

Some HCPs noted that when calculating the price of IV iron, the benefits of avoiding complications of untreated IDA in pregnancy should be considered. This includes the potential ability to avert high fees for hospital admission, preterm delivery and complications of prematurity and postpartum haemorrhage, the cost of which is borne by the health system, women and families, and the whole of society. Although this may be a long-term effect as the benefits of being treated with IV iron, they stated that it should be considered if found to be cost-effective.

### Existing trust between pregnant women and HCPs can avert misconceptions of IV iron therapy

According to all stakeholder groups, the high existing levels of trust between pregnant women and HCPs can avert misconceptions about IV iron therapy.

#### Pregnant women trust HCPs, but vulnerable to misconceptions

Pregnant women felt their trust towards the HCPs was strong and facilitated their belief and acceptance of any form of information or intervention shared by these HCPs. Furthermore, health talks in the antenatal clinic have been and continue to be an effective opportunity to educate pregnant women on health, including the benefits of IV iron therapy.

*“During antenatal, they will have to talk about it to us pregnant women. So, you know, we quickly believe our nurses when they tell us things. So, if you ask them to include the importance of this treatment in the health talk and when they tell us, we will believe that this thing will work, and people will go for it (IV iron).” 37-year-old, Multigravida, Lagos (KII-04)*

The HCP also echoed the above opinions of pregnant women by emphasising the importance of building on this trust when educating them on the importance of IV iron to their health. Furthermore, they stated that the environment should be friendly to gain more trust, enhance assurance and allay fears.

Some pregnant women, HCPs, and domestic decision-makers identified some factors that would prevent pregnant women from receiving IV iron. For example, fear of needles and pain was a significant deterrent for some. However, according to a pregnant woman with anaemia, she “*overcame the fear of needles with the help of a nurse and would be willing to take IV iron*”. The existing relationship with the nurse allowed the woman to understand its importance to her and her baby.

Fear of adverse events (e.g., fever, pruritus, weakness, dizziness, hypotension, myalgia) related to IV iron was another factor that could impede acceptability and use mentioned by pregnant women and HCPs. Generally, respondents had concerns about the potential risks of allergy and adverse events. They felt that adequate information on IV iron, its benefits and the likely adverse events that could occur during its administration would help minimise these concerns. However, according to them, this can only work based on the existing trust.

When probed further, the pregnant women, HCPs, and the domestic decision-makers stated that IV iron is vulnerable to misconceptions, despite the existing trust. According to a male partner in an FGD, who described religion, cultural or traditional beliefs as a strong influence on attitude to health care services stated that “*some people have this belief that herbs and leaves are the best, and there is nothing anyone can say about orthodox medicine that will change their mind…*”. Additionally, for some HCPs, suspicion can be from ignorance. According to them, “*even with this family planning method-Jadelle and Implanon (inserted surgically under the skin), we had many challenges with its uptake within the community*”. To these respondents, these factors could enhance the reluctance and resistance of people to try new treatments, such as IV iron therapy. Furthermore, when sharing their experiences, they likened the vulnerability of IV iron to the misconception people had about the Corona virus disease (COVID-19) vaccine. In an FGD with the HCPs, one of them stated:

*“Our main challenge will be from the current rollout of the COVID-19 vaccine. People will be asking why this iron injection was not introduced until now that they are rejecting the Corona vaccine. They will think we only devised a way of forcing the vaccine on them. And they will say, "we were never given an iron injection before, but now that we are rejecting this vaccine, they now brought it in another way claiming to be for treatment of anaemia so that they will be killing our babies”.” HCP, Female, Kano (FGD P7)*

## Discussion

### Summary of findings

This study explored the prospective acceptability of IV iron for the treatment of IDA in pregnancy among relevant stakeholders in Nigeria. Three main themes and eight sub-themes were linked to the seven constructs of the theoretical framework of acceptability (TFA). In general, respondents expressed positive affective attitudes to IV iron therapy. However, being a facility-based intervention, there is an opportunity cost to receive or administer it. Additionally, the effectiveness of IV iron has been perceived to reduce complications and associated higher fees. However, additional efforts are needed to increase intervention coherence and self-efficacy, which will align with the ethicality of some religious groups.

### Perceived comparative advantages of IV iron are critical for acceptability

In our study, critical to the acceptability of IV iron are the perceived comparative advantages over alternative oral therapy by all stakeholders. One of the advantages is IV iron as a single-dose therapy. Their disposition to IV iron in this regard is understandable for pregnant women. The convenience of receiving IV iron within a few minutes rather than the daily oral use for the whole period of pregnancy and puerperium makes it an appealing option. Even though the respondents answered hypothetically, their positive attitude to IV iron being a single-dosed treatment was similar to that of the participants in a qualitative study from Malawi who had received it [19].

Despite the positive affective attitudes among all stakeholders, IV iron comes with *opportunity cost* for pregnant women and HCPs, centred around IV iron as an HCP-administered intervention. When considered from the perspective of pregnant women, coming to the facility to receive IV iron was deemed a drawback. On the other hand, for the HCPs, it is the time it will take to administer it. Therefore, to receive or administer IV iron as a user or an HCP, some benefits, values, or time must be given up.

Nevertheless, IV iron was *perceived* as an *effective* treatment by pregnant women to overcome the challenge of intolerance, which has been shown to affect about a third of women due to significant gastrointestinal upset and side effects associated with oral iron [42]. Furthermore, the HCPs’ *perceived effectiveness* stems from their experience with the challenges of managing complicated anaemia, which usually presents as an emergency with symptoms such as fatigue, dizziness, palpitations, and breathlessness, amongst others [43, 44]. To them, IV iron can address the challenge of non-adherence, reduce complications, ease the workload related to managing these emergencies and have the capacity to avert higher fees from complicated IDA. In addition, according to Manda-Taylor et al., the outcome of treatment with IV iron could result in good feto-maternal effects [19].

In our study, the HCPs felt that the *ethicality* of IV iron aligned with some religious faiths. An example is the Jehovah’s Witness, whose health-seeking behaviour is influenced by their unique religious beliefs, such as refusal of blood and blood products, putting them at a high risk of maternal morbidity and mortality [45]. The perception of the HCPs stems from the challenges of managing this group of women which often requires critical decision-making, especially in cases of symptomatic IDA necessitating an urgent blood transfusion. However, because of the aversion of these women to blood, IV iron will be an alternative form of treatment to rapidly correct IDA before the need for blood transfusion arises [46–48]. It is important to note that while this affects a small group, it will nevertheless have a significant impact in facilitating the use of IV iron.

### Existing infrastructure in the health facility could be leveraged and strengthened to sustainably provide IV iron

Our findings suggested that the additional *effort* needed to enhance the acceptability of IV iron is to integrate its information into routine antenatal health talk. This health talk is an avenue designed to educate pregnant women on various health issues. Therefore, it can be utilised to inform women about how IV iron works, the indication of use, and its advantages and benefits. And as stated by the HCPs in a study by Mayson E, the resultant effect will facilitate awareness, stimulate *intervention coherence*, and the *self-efficacy* required for pregnant women to receive IV iron therapy [49].

As stated by the HCPs in our study, a lack of confidence in knowledge and skills to administer IV iron therapy could affect its acceptability. Fear of adverse reactions by the HCPs could feed into their lack of confidence. Additionally, sub-optimal knowledge and minimal experience with newer formulations of IV iron therapy might have contributed to their lack of confidence [50]. Fortunately, several studies in HICs and some LMICs have shown that IV iron has an increased safety profile and efficacy over oral iron [50–52]. These findings would reassure the HCPs, enhance self-efficacy, and reduce the restriction to widespread utilisation of IV iron. And as observed in our study, the HCPs were confident they could safely administer IV iron if adequately trained. This training could be aided by providing a guide or protocol, as was done in Malawi [19], to increase their *self-efficacy*.

According to HCPs and facility managers engaged in our study, local health system infrastructure (space), (human) resources and supplies are currently insufficient to implement IV iron. Hence, IV iron’s acceptability will depend on the supply (functioning health system infrastructure) and demand from pregnant women and HCPs. Generally, the existing healthcare system in Nigeria is faced with challenges centred around the supply and demand of healthcare services, necessitating additional efforts for adequate infrastructure and resources [53–56]. These *efforts* could reduce the effect of opportunity cost perceived as associated with receiving or administering IV iron by pregnant women and the HCPs. And hence, improve their *self-efficacy* and their *affective attitudes* in general.

The study results also showed that potentially high out-of-pocket costs could foster a negative *affective attitude* towards IV iron. There were some perceptions that pregnant women with IDA are typical of low socio-economic status. These pregnant women have a high risk for poor nutrition, which can lead to insufficient intake of iron-containing diet [4] and further expose them to its burdens and complications, worsening their economic status [57, 58]. Should the IV iron treatment result in a higher out-of-pocket cost for women, this could prevent equitable distribution, lead to a higher cost burden, and impact their perception of acceptability. Therefore, there is a need for collective *efforts* by the government, in conjunction with health insurance companies, pharmaceutical companies for mass production locally and marketers of the medicine for negotiation. These efforts might ensure the intervention is of low cost to reduce the burden a high price could impose on pregnant women and their families. Additionally, these efforts will improve the *self-efficacy* of these pregnant women and domestic decision-makers to receive this intervention because it is now of low cost and affordable to procure. As for the HCPs who manage them, it will be easier to counsel pregnant women to opt for this form of care as an intervention.

### Existing trust between pregnant women and HCPs can avert misconceptions of IV iron therapy

Leveraging the existing trust between the women and their HCPs could facilitate the *affective attitude* of IV iron. As demonstrated in the study, building on the current trust is an invaluable factor that could allow fruitful discussion and understanding of IV iron therapy, including the process involved with its administration. It could also be utilised to reduce or dispel the fears of needles and adverse events raised by the women in our study. Nevertheless, it is essential to note that despite this trust, IV iron is vulnerable to misconception. A classic example, given by the HCPs in our study, is the seeming similarity between the COVID-19 vaccine and IV iron therapy, both being a new parenteral form of intervention. As already known in the literature, including an observational study by Daniel Kwasi et al. in Iran, COVID-19 is associated with many controversies [59]. Therefore, IV iron, an unfamiliar therapy to pregnant women in general, could easily be misinterpreted as another type of COVID-19 vaccine deliberately introduced to enforce and increase its uptake. Other examples are the superstitious belief that IV iron can lead to miscarriage [19] and its association with severe hypersensitivity reactions [43]. Therefore, to mitigate this misconception, additional *efforts* are needed to reinforce the HCP-patient interaction with appropriate health communication [60].

### Interwoven relationships between the TFA constructs and acceptability of IV iron therapy

**Fig. 1:**
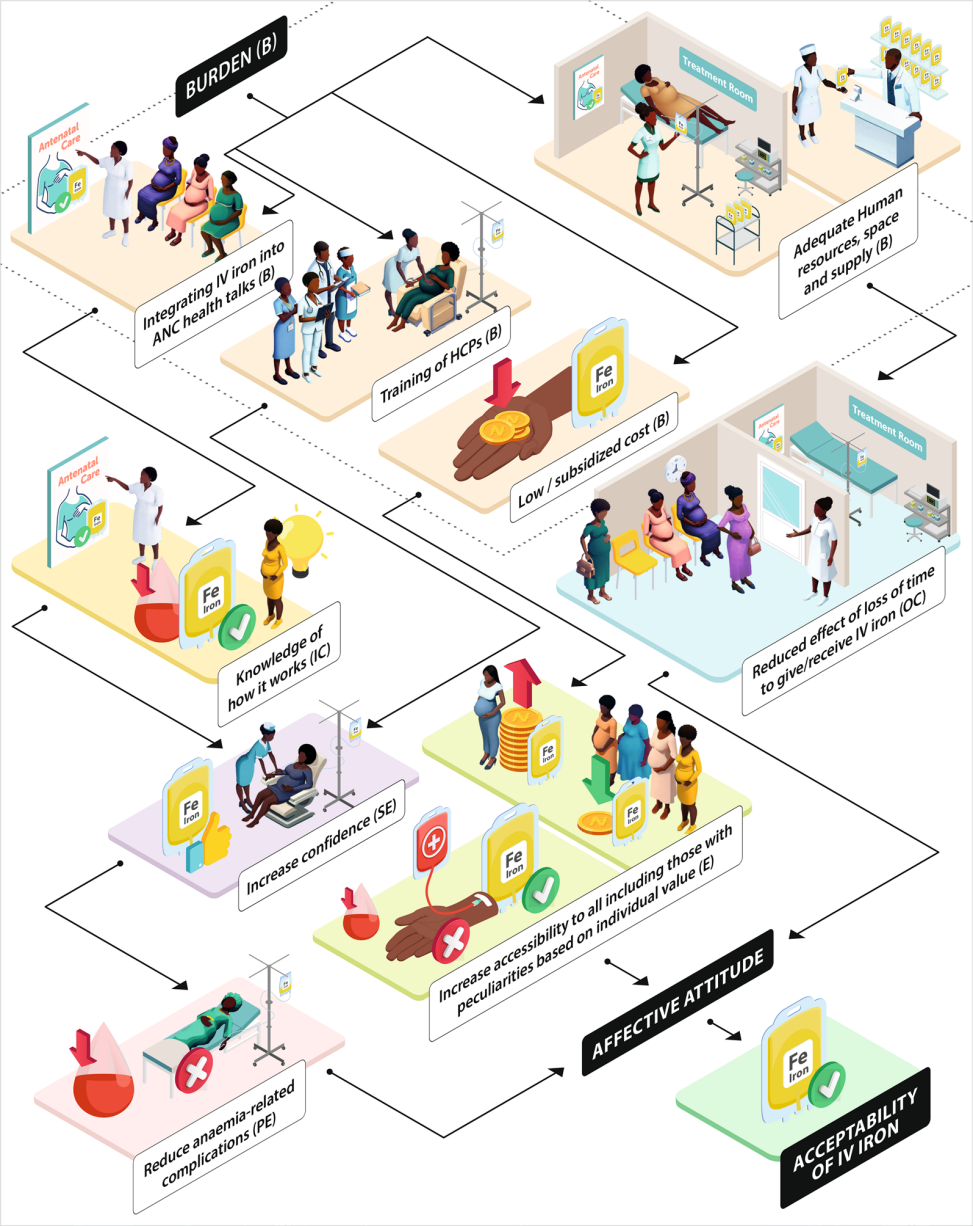
Illustrating the interwoven relationships between the constructs of the theoretical framework of acceptability and the acceptability of IV iron B burden, IC intervention coherence, SE self-efficacy, OC opportunity cost, PE perceived effectiveness, HCP health care personnel, IV intravenous iron

As revealed in our study, the responses elicited from the stakeholders gave more profound insights into the critical role the TFA constructs played in understanding the acceptability of IV iron therapy in our setting. As illustrated in Fig. 1, instituting additional efforts (burden) into the present healthcare system will trigger cascades of TFA constructs with the resultant effects of positive affective attitudes and the acceptability of IV iron therapy. For example, integrating IV iron into routine ANC practices (burden) will enhance the knowledge of how it works (intervention coherence), increase the confidence to receive or administer it (self-efficacy) and reduce anaemia-related complications (perceived effectiveness). Furthermore, training of HCPs (burden) will enhance confidence (self-efficacy) to give IV iron therapy. At the same time, adequate human resources, spaces, and constant supply (burden) will reduce the time and burden perceived to be associated with receiving or administering IV iron therapy (opportunity cost). In addition, the low/subsidised cost of IV iron (burden) will facilitate easy access to the intervention for all pregnant women, including those with peculiarities based on their ethicality. Therefore, resulting in positive affective attitudes among the users and the givers of IV iron and facilitate its acceptability.

### Implications for practice, policy, and future research

There are clear revealed interwoven relationships between insights gathered from participants in our study and the constructs of TCA, which ultimately would contribute to increasing the acceptability of IV iron (Fig. 1). Considered together, these are the implications for practice and policy of our research and explain the perceived modalities and strategies needed for the acceptability and implementation of IV iron, keeping in mind the peculiarities of its challenges in resource-limited settings like ours. Firstly, targeted education, effective communication, and dissemination are necessary to facilitate intervention coherence in antenatal care practices. This would also accelerate confidence and dispel the fears and restrictions preventing IV iron’s widespread use in Nigeria [51]. Secondly, investing in the training of HCPs physically or virtually, through workshops or seminars, in addition to developing a protocol, is expedient to guide clinical decision-making. This would enhance the intervention coherence as well as the self-efficacy of HCPs [19, 49] Thirdly, considering the complexity of the process of IV iron administration, it is imperative to make this process seamless for its feasibility in our healthcare system with consideration for the needs at different levels of care. To do so, the government needs to provide adequate human resources to reduce the workload perceived by the HCPs as an impediment to IV iron therapy's acceptability and perhaps with consideration for task sharing and or shifting among the HCPs [62]. Additionally, there should be provision of dedicated spaces to receive and administer this treatment comfortably. Examples of such areas are the emergency department [61], the wards and on an outpatient basis [62, 63]. Lastly, administering a single-dosed IV iron could abate the cost implication from repeated use of oral iron and reduce the risk of morbidity and mortality to both the mother and the baby [19, 51]. Another solution, as proffered by the study populace, was subsidising IV iron, which has been described as actions needed to facilitate equitable distribution to all. The health insurance scheme was an example postulated by the HCPs for subsidising the cost. However, it still needs to be universally available in Nigeria [64, 65]. Therefore, efforts should be made to integrate community-based health insurance programmes into Nigeria’s present health care insurance scheme for easy access to IV iron by pregnant women who need it more to benefit immensely from it [65].

Findings from our study also generate implications for future research as it would be expedient to carry out a real-world implementation study. Even though our findings identified some potential factors that could affect the acceptability of IV iron, it is necessary to explore and ascertain the actual status of these factors as barriers and facilitators in real-world settings. This is necessary to monitor the process of defining the complete pathway of screening, diagnosis, and treatment of IDA in pregnancy, focusing on intravenous iron use. This should be outside of the controlled settings of a clinical trial, with stakeholders that are not part of an ongoing trial [66].

### Strengths and limitations

To the best of our knowledge, this is the first study in Nigeria to explore the acceptability of IV iron among multiple key stakeholder groups with varying perspectives. The use of the well-structured multifaceted TFA offered the possibility to explore and understand the perception of these diverse stakeholders on the potential acceptability of IV iron therapy. In addition, the large sample size recruited for the study, the appropriateness of which was assessed with the principle of information power, offered us an opportunity to have comprehensive narratives that enabled data and analytical sufficiency [67]. Furthermore, we established trustworthiness through a transparent process throughout the study period and ensured a rigorous analytical approach to enhance credibility and facilitate reflexivity. On the other hand, the exploration of the acceptability of IV iron during the pre-intervention phase of the trial could be considered a limitation of our study. The responses elicited were more from a perceived point of view, which might not accurately convey their perception. Nevertheless, they could relate their perception with their experiences based on their respective capacity as providers or users of IV iron and as people who support the users. Furthermore, the use of multiple interviewers/moderators could be a source of interviewer bias. However, our implementation of a standard operating protocol for all interviewers and moderators was aimed at minimising this risk. In addition, cross-lingual interviews and transcription pose challenges, leading to the possibility of missing some nuances. Therefore, to ensure trustworthiness, the recordings were subjected to forward translation, back-translation, and transcription by skilled bilingual research assistants with a subset verified by a professional translator not part of the research team.

## Conclusion

Our study explored the acceptability of IV iron therapy as part of formative research before the onset of the randomised clinical trial. IV iron has a high potential to be the preferred treatment for IDA in pregnancy in Nigeria if proven effective. Key issues which need to be considered to improve the acceptability of this treatment include HCP training, optimisation of information delivery and clinical care provision during antenatal visits, uninterrupted supply of medicine and supplies, and subsidies to offset the expected higher price. The interwoven nature of these issues was clearly depicted in a model which could facilitate the exploration of similar interventions to serve as a guide with ease of use to produce in-depth narratives and meaning to the intervention of interest regarding acceptability. Further research to facilitate the routine provision of IV iron therapy will likely enable rapid scale-up of this intervention to address a significant public health problem in Nigeria.

## Data Availability

The datasets used and/or analysed during the current study are available from the corresponding author on reasonable request.

## Abbreviations

AIP: Anaemia in pregnancy
LMIC: Low and middle-income countries
IDA: Iron deficiency anaemia
WHO: World Health Organization
IV: Intravenous
HICs: High-income countries
IVON: IV versus oral iron for Iron Deficiency Anaemia in Pregnant Nigerian Women
COREQ: Consolidated criteria for reporting qualitative research
PHC: Primary health centres
HIV: Human immunodeficiency virus
HCP: Healthcare providers
FGDs: Focus group discussions
KIIs: Key informant interviews
TFA: Theoretical framework of acceptability

## References

[1] Karami M, Chaleshgar M, Salari N, Akbari H, Mohammadi M (2022). Global prevalence of anemia in pregnant women: a comprehensive systematic review and meta-analysis. Matern Child Health J.

[2] Ugwu NI, Uneke CJ (2020). Iron deficiency anemia in pregnancy in Nigeria-a systematic review. Niger J Clin Pract.

[3] World Health Organization. Nutritional anaemias: tools for effective prevention and control. 2017. [cited 2022 Nov 13]. https://apps.who.int/nutrition/publications/micronutrients/anaemia_iron_deficiency/WHO_NHD_01.3/en.

[4] Oyelese AT, Ogbaro DD, Wakama TT, Adediran A, Gbadegesin A, Awodele IO, et al. Socio- Economic determinants of prenatal anaemia in rural communities of South-West Nigeria: a preliminary report. Am J Blood Res. 2021;11(4). PMC8446826 34540350

[5] Sholeye O, Animasahun V, Shorunmu T (2017). Anemia in pregnancy and its associated factors among primary care clients in Sagamu, Southwest, Nigeria: a facility-based study. J Family Med Prim Care..

[6] Kang SY, Kim HB, Sunwoo S (2020). Association between anemia and maternal depression: a systematic review and meta-analysis. J Psychiatric Res.

[7] Ramanathan GAS (2006). Postpartum hemorrhage. J Obstet Gynecol Can.

[8] Christian P. Nutrition and maternal survival in developing countries. Handbook of nutrition and pregnancy. 2008. 10.1007/978-1-59745-112-3_21.

[9] Daru J, Zamora J, Fernández-Félix BM, Vogel J, Oladapo OT (2018). Risk of maternal mortality in women with severe anaemia during pregnancy and postpartum: a multilevel analysis. Lancet Glob Health.

[10] World Health Organization. WHO recommendations on antenatal care for a positive pregnancy experience. 2016. [cited 2022 Nov 9]. Available from: https://www.who.int/publications-detail-redirect/9789241549912. 28079998

[11] Balarajan Y, Ramakrishnan U, Özaltin E, Shankar AH, Subramanian SV (2011). Anaemia in low- income and middle-income countries. The Lancet.

[12] Dhanani JV, Ganguly BP, Chauhan LN (2012). Comparison of efficacy and safety of two parenteral iron preparations in pregnant women. J Pharmacol Pharmacother.

[13] Tolkien Z, Stecher L, Mander AP, Pereira DIA, Powell JJ. Ferrous sulfate supplementation causes significant gastrointestinal side-effects in adults: a systematic review and meta- analysis. PLoS ONE. 2015; 10. 10.1371/journal.pone.0117383 PMC4336293 25700159

[14] Pavord S, Daru J, Prasannan N, Robinson S, Stanworth S, Girling J (2020). UK guidelines on the Management of iron deficiency in pregnancy. Br J Haematol.

[15] FIGO Working Group on Good Clinical Practice in Maternal-Fetal Medicine (2019). Good clinical practice advice: Iron deficiency anemia in pregnancy. Int J Gynecol Obstet.

[16] Auerbach M, Gafter-Gvili A, Macdougall IC (2020). Intravenous iron: a framework for changing the management of iron deficiency. Lancet Haematol.

[17] Radhika AG, Sharma AK, Perumal V, Sinha A, Sriganesh V, Kulshreshtha V (2019). Parenteral versus oral iron for treatment of iron deficiency anaemia during pregnancy and post- partum: a systematic review. J Obstet Gynecol India..

[18] Sekhon M, Cartwright M, Francis JJ (2017). Acceptability of healthcare interventions: an overview of reviews and development of a theoretical framework. BMC Health Serv Res.

[19] Manda-Taylor L, Kufankomwe M, Chatha G, Chipeta E, Mamani-Mategula E, Mwangi MN (2022). Perceptions and experiences of intravenous iron treatment for anaemia in pregnancy in Malawi: a formative qualitative study. Gates Open Res..

[20] Creswell JW, Hanson WE, Clark Plano VL, Morales A (2007). Qualitative research designs: selection and implementation. Couns Psychol.

[21] Creswell JW, Poth CN. Qualitative inquiry & research design; Choosing among five approach. SAGE Publications, Inc. 2018. https://us.sagepub.com/en-us/nam/qualitative-inquiry-and-research-design/book246896.

[22] Afolabi BB, Babah OA, Akinajo OR, Adaramoye VO, Adeyemo TA, Balogun M (2022). Intravenous versus oral iron for iron deficiency anaemia in pregnant Nigerian women (IVON): study protocol for a randomised hybrid effectiveness-implementation trial. Trials.

[23] Tong A, Sainsbury P, Craig J (2007). Consolidated criteria for reporting qualitative research (COREQ): a 32-item checklist for interviews and focus groups. Int J Qual Health Care.

[24] National Population Commission Nigeria. National Demographic and Health Survey. Nigeria 2018. The DHS Program. 2018;1–748. [cited 2022 Sep 17]. Available from: https://dhsprogram.com/pubs/pdf/F.

[25] World Health Organization. Trends in maternal mortality 2000 to 2020: estimates by WHO, UNICEF, UNFPA, World Bank Group and UNDESA/Population Division. 2023 [cited 2023 Mar 18]; Available from: https://www.who.int/publications-detail-redirect/9789240068759.

[26] United Nations. World Population Prospects 2019: Data Booklet. Department of Economic and Social Affairs Population Division. 2019; https://population.un.org/wpp/publications/files/wpp2019_databooklet.pdf.

[27] Yar I, Said I, Yar IS (2013). Knowledge and barriers in utilization of maternal health care services in Kano state, Northern Nigeria. Eur J Biol Med Sci Res..

[28] Ademuyiwa IY, Faronbi J, Oyediran OO, Erondu C (2020). Antenatal care services utilization and Factors influencing it among pregnant women in a Teaching Hospital in Lagos, Nigeria. Trop J Health Sci.

[29] Nwizu EN, Iliyasu Z, Ibrahim SA, Galadanci HS. Socio-demographic and maternal factors in anaemia in pregnancy at booking in Kano, northern Nigeria. Afr J Reprod Health. 2011;15(4). 22571103

[30] Erhabor O, Muhammad AD, Adias TC, Ahmed Y, Erhabor T (2020). Anaemia and thrombocytopenia among pregnant women attending Aminu Kano Teaching Hospital, Kano State, North Western Nigeria. Hum Antibodies.

[31] Olukosi AY, Olakiigbe A, Ajibaye O, Orok BA, Aina OO, Akindele SK (2020). Socio-economic behavioural indicators of falciparum malaria parasitaemia and moderate to severe anaemia among pregnant women attending antenatal clinics in Lagos, Southwest Nigeria. Malar J.

[32] Anorlu RI, Oluwole AA, Abudu OO (2006). Sociodemographic factors in anaemia in pregnancy at booking in Lagos, Nigeria. J Obstet Gynaecol (Lahore).

[33] Ademiluyi IA, Aluko-Arowolo SO (2009). Infrastructural distribution of healthcare services in Nigeria: an overview. J Geography Regional Planning.

[34] World Health Organization 2018. Health situation. 2018 [cited 2022 Sep 17]; Available from: http://apps.who.int/gho/data/node.cco.

[35] Farrugia B (2019). WASP (write a scientific paper): sampling in qualitative research. Early Hum Dev.

[36] Malterud K, Siersma VD, Guassora AD (2016). Sample size in qualitative interview studies: guided by information power. Qual Health Res.

[37] Moen K, Middelthon AL. Qualitative research methods. Research in medical and biological sciences: from planning and preparation to grant application and publication. 2015;321–78. https://www.researchgate.net/publication/283133114_Qualitative_Research_Methods.

[38] McGrath C, Palmgren PJ, Liljedahl M (2019). Twelve tips for conducting qualitative research interviews. Med Teach.

[39] Graneheim UH, Lundman B (2004). Qualitative content analysis in nursing research: concepts, procedures and measures to achieve trustworthiness. Nurse Educ Today.

[40] Hsieh HF, Shannon SE (2005). Three approaches to qualitative content analysis. Qual Health Res.

[41] Assarroudi A, Heshmati Nabavi F, Armat MR, Ebadi A, Vaismoradi M (2018). Directed qualitative content analysis: the description and elaboration of its underpinning methods and data analysis process. J Res Nurs.

[42] Khalafallah A, Dennis A, Bates J, Bates G, Robertson IK, Smith L (2010). A prospective randomized, controlled trial of intravenous versus oral iron for moderate iron deficiency anaemia of pregnancy. J Intern Med.

[43] Garzon S, Cacciato PM, Certelli C, Salvaggio C, Magliarditi M, Rizzo G (2020). Iron deficiency anemia in pregnancy: novel approaches for an old problem. Oman Med J.

[44] Benson CS, Shah A, Frise MC, Frise CJ (2021). Iron deficiency anaemia in pregnancy: a contemporary review. Obstet Med.

[45] Van Wolfswinkel ME, Zwart JJ, Schutte JM, Duvekot JJ, Pel M, van Roosmalen J (2009). Maternal mortality and serious maternal morbidity in Jehovah’s witnesses in the Netherlands. BJOG.

[46] Zeybek B, Childress AM, Kilic GS, Phelps JY, Pacheco LD, Carter MA (2016). Management of the Jehovah’s witness in obstetrics and gynecology: a comprehensive medical, ethical, and legal approach. Obstet Gynecol Surv.

[47] DeLoughery TG (2020). Transfusion replacement strategies in Jehovah’s witnesses and others who decline blood products. Clin Adv Hematol Oncol.

[48] RCOG. Blood Transfusion in Obstetrics. 2015. https://www.rcog.org.uk/media/sdqcorsf/gtg-.pdf.

[49] Mayson E, Ampt AJ, Shand AW, Ford JB (2016). Intravenous iron: barriers and facilitators to its use at nine maternity hospitals in New South Wales, Australia. Aust N Z J Obstet Gynaecol.

[50] Esen UI (2017). Iron deficiency anaemia in pregnancy: the role of parenteral iron. J Obstet Gynaecol (Lahore).

[51] Divakar H (2012). Iron-deficiency anemia in pregnant women: what preventing practitioners from using IV iron sucrose. Int J Infertility Fetal Med.

[52] Auerbach M, Macdougall IC (2014). Safety of intravenous iron formulations: facts and folklore. Blood Transfus.

[53] Udenigwe O, Okonofua FE, Ntoimo LFC, Imongan W, Igboin B, Yaya S (2021). Perspectives of policymakers and health providers on barriers and facilitators to skilled pregnancy care: findings from a qualitative study in rural Nigeria. BMC Pregnancy Childbirth.

[54] Muhammed KA, Umeh KN, Nasir SM, Suleiman IH (2013). Understanding the barriers to the utilization of primary health care in a low-income setting: Implications for health policy and planning. J Public Health Afr.

[55] Olutuase VO, Iwu-Jaja CJ, Akuoko CP, Adewuyi EO, Khanal V (2022). Medicines and vaccines supply chains challenges in Nigeria: a scoping review. BMC Public Health.

[56] Siekmans K, Roche M, Kung’u JK, Desrochers RE, De-Regil LM (2018). Barriers and enablers for iron folic acid (IFA) supplementation in pregnant women. Matern Child Nutr.

[57] Annamraju H, Pavord S (2016). Anaemia in pregnancy. Br J Hosp Med.

[58] Lopez A, Cacoub P, Macdougall IC, Peyrin-Biroulet L (2016). Iron deficiency anaemia. The Lancet.

[59] Ahorsu DK, Lin CY, Yahaghai R, Alimoradi Z, Broström A, Griffiths MD (2022). The mediational role of trust in the healthcare system in the association between generalized trust and willingness to get COVID-19 vaccination in Iran. Hum Vaccin Immunother.

[60] Nganga SW, Otieno NA, Adero M, Ouma D, Chaves SS, Verani JR (2019). Patient and provider perspectives on how trust influences maternal vaccine acceptance among pregnant women in Kenya. BMC Health Serv Res.

[61] Beverina I, Razionale G, Ranzini M, Aloni A, Finazzi S, Brando B (2020). Early intravenous iron administration in the Emergency Department reduces red blood cell unit transfusion, hospitalisation, re-transfusion, length of stay and costs. Blood Transfus.

[62] Barrera M and SD. Implementation of an outpatient iron infusion clinic: alternative approach to treat iron deficiency anemia in pregnancy. 2020. Nicole Wertheim College of Nursing Student Projects. 4. Available from: https://digitalcommons.fiu.edu/cnhs-studentprojects/4.

[63] Kriplani A, Mahey R, Dash BB, Kulshreshta V, Agarwal N, Bhatla N (2013). Intravenous iron sucrose therapy for moderate to severe anaemia in pregnancy. Indian J Med Res.

[64] Alawode GO, Adewole DA (2021). Assessment of the design and implementation challenges of the National Health Insurance Scheme in Nigeria: a qualitative study among sub-national level actors, healthcare and insurance providers. BMC Public Health.

[65] Odeyemi IA (2014). Community-based health insurance programmes and the national health insurance scheme of Nigeria: challenges to uptake and integration. Int J Equity Health.

[66] Eboreime E, Banke-Thomas A, Obi-Jeff C, Adelabu Y, Balogun M, Aiyenigba AA (2023). A continuous quality improvement strategy to strengthen screening practices and facilitate the routine use of intravenous iron for treating anaemia in pregnant and postpartum women in Nigeria: a study protocol. Implement Sci Commun..

[67] LaDonna KA, Artino AR, Balmer DF (2021). Beyond the Guise of Saturation: Rigor and Qualitative Interview Data. J Graduate Med Educ NLM (Medline).

